# Treatment of T2DM-related inflammation and vascular injury by regulating cellular crosstalk in the islet microenvironment

**DOI:** 10.3389/fendo.2025.1551853

**Published:** 2025-10-13

**Authors:** Aru Sun, Haoyu Yang, Jun Sun, Jinli Luo, Ling Zhou, Tingting Bao, Xiaolin Tong, Yiqun Lin, Lin Han

**Affiliations:** ^1^ School of Traditional Chinese Medicine, Changchun University of Chinese Medicine, Changchun, China; ^2^ School of Traditional Chinese Medicine, Binzhou Medical University, Yantai, China; ^3^ School of Traditional Chinese Medicine, Bozhou University, Bozhou, China; ^4^ China Traditional Chinese Medicine Holdings Co Limited, Guangdong e-fong Pharmaceutical CO., LTD., Foshan, Guangdong, China; ^5^ School of Clinical Medicine, Chengdu University of Chinese Medicine, Chengdu, China; ^6^ Institute of Metabolic Diseases, Guang’anmen Hospital, China Academy of Chinese Medical Sciences, Beijing, China; ^7^ Department of Endocrinology, Guang’anmen Hospital South Campus, China Academy of Chinese Medical Sciences, Beijing, China

**Keywords:** type 2 diabetes mellitus, islet microenvironment, cellular crosstalk, natural products, therapy

## Abstract

Type 2 diabetes mellitus (T2DM), a complex systemic metabolic disorder caused by multiple factors, has been linked to numerous acute and chronic complications. T2DM pathogenesis includes glucotoxicity, lipotoxicity, inflammatory cytokines, and amyloid formation. Within the pancreatic islet microenvironment, the crosstalk among cell types plays a significant role in these pathogenic mechanisms. Islet β cells, macrophages, and endothelial cells, the three primary cell types, engage in intercellular communication under physiological and pathological conditions, critical to maintaining islet homeostasis and promoting the pathological progression of T2DM. This review discusses the interactions between these islet cells, particularly how their crosstalk affects islet function and T2DM development. Additionally, natural products targeting islet cell interactions are discussed as a therapeutic approach for T2DM, along with other personalized treatment options, including exosomes, parasitic therapy, and dietary interventions. Emerging strategies that regulate intercellular signaling and complex crosstalk within the islet microenvironment offer promising avenues for T2DM treatment.

## 1 Introduction

Type 2 diabetes mellitus (T2DM) comprises 90% of the global diabetes cases, posing severe threats to the life and socioeconomic well-being worldwide (1, 2). In 2021, the International Diabetes Federation (IDF) estimated the global prevalence of diabetes at 537 million individuals. This number is projected to rise to 643 million by 2030 and 783 million by 2045 (3). Despite these alarming figures, diabetes is often overlooked and underestimated as a cause of mortality in routine health statistics. Between 2000 and 2019, diabetes was responsible for a 0.14-year reduction in health-adjusted life expectancy (HALE) among individuals aged 30 (4). The latest edition of the IDF Diabetes Atlas (11th edition) reports that, in 2024, diabetes-related deaths exceeded 3.4 million, accounting for 9.3% of total global mortality (http://www.diabetesatlas.org/). Notably, many individuals with undiagnosed diabetes already exhibit complications such as chronic kidney disease, heart failure, retinopathy, and neuropathy. Macrovascular and microvascular complications associated with T2DM are the primary causes of mortality and disability in affected individuals (5, 6), with cardiovascular disease being the leading cause of death (7).

The pathophysiological changes in T2DM are primarily characterized by β-cell dysfunction, insulin resistance, and chronic inflammation, all of which impede glycemic control (5, 8). For instance, excessive lipid accumulation disrupts insulin signaling in cardiomyocytes, leading to cardiac insulin resistance and the activation of profibrotic pathways that promote myocardial fibrosis and exacerbate diastolic dysfunction (9). In diabetic nephropathy, hyperglycemia induces the formation of advanced glycation end products, activates protein kinase C, increases the expression of transforming growth factor β (TGF-β) and GTP-binding proteins, and generates reactive oxygen species (ROS), all of which contribute to various types of renal cell injury (10). The excessive accumulation of ROS can trigger mitochondrial damage in retinal cells, apoptosis, inflammatory responses, lipid peroxidation, and alterations in retinal structure and function (11). Additionally, hyperglycemia, dyslipidemia, and insulin resistance induce oxidative stress, mitochondrial dysfunction, and inflammation, resulting in neuronal and Schwann cell injury and demyelination (12). In summary, oxidative stress, inflammatory responses, and endothelial dysfunction induced by hyperglycemia form the common pathophysiological basis for these complications.

In addition, glucose toxicity, lipotoxicity, fatty acids, inflammatory cytokines, and amyloid formation related to β-cell pathology have been implicated in its progression (13). Notably, immune-cell infiltration and amyloid deposition during islet inflammation are key contributing factors that lead to islet fibrosis (14). The islet is a highly vascularized and innervated complex structure comprising islet endocrine cells, islet endothelial cells, islet macrophages, and an extracellular matrix (ECM). Although macrophage infiltration is observed in the insulitis of type 1 diabetes, it is more pronounced in the insulitis of T2DM and has been confirmed in human T2DM islet pathology (15, 16). In response to pathological stimulation caused by T2DM, the islet must trigger stress responses and defense mechanisms. This includes expanding β-cell clusters, allowing macrophages to infiltrate the islet, and involving vascular endothelial processes. Involved β-cell inflammatory pathways include Toll-like receptor (TLR) signaling, the nuclear factor kappa B (NF-κB) pathway, and NLRP3/caspase 1 (17).

Within the islet microenvironment, the islet cell distribution in close contact with each other facilitates cell-to-cell crosstalk. Changes in the polarization and recruitment of islet macrophages contribute to β-cell dysfunction and islet inflammation (17 **).** Islet macrophages and β cells communicate via autocrine and paracrine signaling, exchanging cytokines, hormones, neurotransmitters, and other information to mediate physiological balance and pathological inflammatory responses. Recent research has mainly focused on how islet-cell crosstalk promotes β-cell proliferation, functional recovery, and therapeutic effects in diabetes (18–21). Additionally, islet endothelial cells, an important part of the islet vascular system, are key in maintaining normal islet function, microenvironmental homeostasis, and the pathology of T2DM through intercellular interactions. In this process, cellular crosstalk mediated by extracellular vesicles and exosomes has gained attention.

Natural products have protective effects against metabolic diseases such as diabetes, hypertension, hyperlipidemia, and cardiovascular diseases. Indeed, certain natural products and their associated active compounds can regulate islet function and mitigate T2DM through various mechanisms. These compounds have structurally diverse and bioactive properties, which provide advantages in managing complex islet pathological processes and regulating intercellular signaling molecules. This review discusses the crosstalk among the three major cell types within the T2DM islet microenvironment—β cells, macrophages, and endothelial cells—and the molecular mechanisms underlying their interactions under pathological conditions and explores how natural products regulate islet-cell crosstalk. Additionally, exosomes, parasitic agents, and dietary adjustments provide further opportunities and insights for treating T2DM via islet cell interactions.

## 2 Crosstalk among islet β cells, islet macrophages, and endothelial cells in the islet microenvironment and their role in T2DM

### 2.1 Crosstalk between β cells and islet macrophages

The role of β cells and islet macrophages in islet homeostasis has been extensively studied. Evidence from rodent models indicates that islet macrophages promote β-cell regeneration. In the islets of mice with β cells overexpressing vascular endothelial growth factor-A (*Vegf-a*), macrophages produce growth factors (*Hgf, Igf1*, and *Pdgfb*) that support β-cell regeneration, along with chemokines associated with tissue repair (*Ccl12* and *Ccl2*), cell adhesion molecules (*Icam1* and *Vcam1*), and metalloproteinases (MMPs) (22). Meanwhile, macrophages are recruited into the islet, which is essential for β-cell proliferation. Platelet-derived growth factor (PDGF) from macrophages is closely associated with angiogenesis and cell proliferation (23, 24). In the islet macrophages of obese mice, PDGF-PDGFR signaling mediates β-cell proliferation (18), which has also been confirmed in human β cells (25). In contrast, adult human islets exhibit a weaker response to PDGF signaling (18). As a member of the CCN family associated with ECM proteins, connective tissue growth factor (CTGF/CCN2) functions as a factor for β-cell proliferation and in recruiting specific immune cells (26). CTGF can promote the proliferation and regeneration of ablated β cells, which involves macrophages (27). Consistent with these findings, in the pancreatic duct ligation model, M2 macrophages in the islets promote β-cell proliferation by releasing growth factors (TGF-β1 and EGF) that upregulate SMAD7 in β cells (28). Therefore, cytokines and chemokines are key to the bidirectional communication between β cells and macrophages.

Gene expression analysis of CTGF-induced isolated mouse islets showed that during β-cell destruction, gene markers associated with the pro-inflammatory M1 macrophage polarization, such as *CD86* and *IL-12b*, were specifically increased. In contrast, the expression of genes related to M2 macrophage polarization, including *Arg1*, *Mgl1*, and *Chil3*, was decreased (27). This suggests that macrophages alter their polarization phenotype in response to β-cell regeneration. Conversely, the presence of macrophages influences β-cell phenotype. Under the same conditions, removing macrophages resulted in a lesser decline in MafA+ β cells compared to β-cell ablation with CTGF alone. This suggests that macrophage depletion promotes a more mature β-cell phenotype (27).

*In vivo and in vitro* studies in T2DM mice have shown that β-cells may be early responders to the induction of chemokine production and the recruitment of M1 pro-inflammatory monocytes/macrophages in the islets by excess saturated fatty acids, such as palmitate (29). Additionally, islet macrophages can sense endogenous ATP signals released by stressed β-cells and become activated (30). In a transgenic mouse model expressing human islet amyloid polypeptide (IAPP), studies have observed that resident islet macrophages exhibit a pro-inflammatory phenotype and mediate IL-1β production in the islets and β-cell dysfunction (31). These findings suggest that the interaction between macrophages and β-cells may accelerate the inflammatory process in the islets. In T2DM, pro-inflammatory cytokines, such as IL-1β, IL-6, and TNF-α, induce β-cell dedifferentiation and impair insulin secretion and islet function (32). In human T2DM studies, previous reports have also shown an increased proportion of islet infiltration by macrophages (15). Like rodent models, the increase in leukocytes in human T2DM islets is closely related to islet dysfunction. However, human model results have also found that, in different pathological states of T2DM with high and low insulin secretion, there are differences in the degree of islet inflammation and the recruitment of leukocytes (33). Moreover, early studies using TNF-α, LPS, and IFN-γ to activate resident islet macrophages have observed their effects on human islet cell function, finding that they can induce the release of IL-1 from human islets and induce β-cell expression of iNOS, increased nitric oxide production, and reduced insulin secretion, leading to β-cell damage (34, 35).

Compared with rodent models, the importance of islet macrophages for β-cell development and maintenance has been similarly confirmed in human models. For example, *OP/OP* mice, which lack macrophages due to a mutation in the *CSF-1* gene, exhibit reduced β-cell mass during both embryonic and adult stages (36). Similarly, during human pancreatic development at 6–12 weeks of gestation, the presence of *CD68+* macrophages and *CSF-1* signaling has been observed (37), indicating that islet macrophages are crucial for the normal development and maintenance of β-cells and the pancreas. Additionally, following apoptotic β-cell death, islet macrophages increase the secretion of insulin-like growth factor-1 (IGF-1) to help β cells maintain the second phase (30 min) insulin secretion (38). Physiological proliferation of β cells has been reported in mice during late pregnancy. Endo et al. investigated the restoration of β-cell numbers and found that, under HTR1D signaling, β cells upregulate CXCL10, activating the CXCL10-CXCR3 axis, which attracts macrophages to the islets. During postpartum, these macrophages phagocytize the excessively proliferated β cells, enabling mice to adapt to the reduced insulin demand following the delivery of offspring (39).

### 2.2 Crosstalk between β cells and islet endothelial cells

Islet endothelial cells form an extensive and tortuous capillary network in a highly fenestrated arrangement (40, 41). The structure of endothelial fenestration and the functional activity of endothelial cells depend on the involvement of β cell-derived VEGF-A (42). VEGF-A signaling coordinates islet vascularization by promoting endothelial cell proliferation (43).

In mouse models with induced VEGF-A overexpression, there is a notable rise in the number of islet endothelial cells, alongside a marked increase in β-cell loss. After VEGF-A levels are normalized, β cells regenerate and largely restore their function through a brief burst of proliferation (22). Thus, VEGF produced by β cells is a key factor influencing islet endothelial cell and β-cell function. Additionally, endothelial cells interact with β1 integrin on β cells via derived factors, such as laminins and collagen type IV (COL-IV), forming the vascular basement membrane, creating a vascular niche to promote β-cell function, insulin gene expression, and islet homeostasis (44). CTGF is another unique derived factor expressed in endothelial and embryonic β cells, regulating embryonic β-cell proliferation through autocrine mechanisms (45). Inactivation of CTGF, whether from endothelial or β cells, disrupts β-cell proliferation (45). Gene expression analysis of isolated mouse islets indicates that the role of CTGF in promoting β-cell proliferation may involve upregulating cell cycle regulators (cyclin D3 and B1), TGF-β signaling, Wnt genes, and other growth factors (hepatocyte growth factor [HGF] and serotonin [5-HT]) (26). Additionally, other factors from islet endothelial cells that promote β-cell proliferation and function include HGF, fibroblast growth factor (FGF), and thrombospondin-1 (THBS1) (46).

Furthermore, damage to islet vascular endothelium can lead to β-cell failure (47). Angiotensin (1–7) is an important vasodilatory regulatory peptide in the renin-angiotensin system (RAS). When administered systemically, it improves islet function in rats with T2DM by binding to the Mas receptor. This promotes the expression of endothelial nitric oxide synthase (eNOS) and the release of nitric oxide (NO) in islet endothelial cells. This improves pancreatic microcirculation, reduces β-cell apoptosis, and enhances insulin secretion (48).

Notably, insulin secreted by β cells can act as a mediator in the islet capillary network, promoting islet blood flow (49). Insulin receptor substrate-2 (IRS2) in islet endothelial cells plays a critical role in glucose-induced insulin secretion and maintaining islet blood flow (50). The molecular signals produced by β cells and islet endothelial cells can mutually influence each other’s cells. A recent study established an *in vitro* co-culture model of βTC6 cells and islet endothelial cells (MS1) and found that knocking down the RNA-binding protein HuD in βTC6 cells inhibited the growth and motility of islet endothelial cells (51). This finding was confirmed in HuD knockout mice, where the number of islet endothelial cells was reduced. This effect is mainly related to the binding of the 3’ untranslated region (UTR) of Col18a1 and Serpin E1 mRNA and increased translation of the *EGFP* reporter gene. This indicates that HuD acts as a translational repressor, negatively regulating angiogenesis-inhibiting factors endostatin and Serpin E1, which aids in modulating the crosstalk between β cells and MS1 cells (51).

### 2.3 Crosstalk between islet macrophages and endothelial cells

Under homeostasis, perivascular macrophages in the islets directly contact endothelial cells, extending partially through the endothelium into the vascular lumen (52). When inflammation is induced by injury or infection, endothelial activation shifts to a pro-adhesive state that recruits immune cells and adhesion molecules (53), primarily mediated by the transcriptional regulatory program of endothelial cells activated by NF-κB (54). Recruited monocytes/macrophages briefly adhere to the endothelial surface before migrating through the vessel wall to the site of injury (55).

The recruited monocytes/macrophages produce pro-angiogenic signals (such as VEGF, ANG-2, and FGF), and endothelial cells respond to surrounding signals, undergoing proliferation and migration (56). VEGF activates the VEGF receptor VEGFR2 on tip cells, stimulating their outward expansion and protrusion, thereby inducing vessel sprouting (57). Subsequently, macrophages interact with the filopodia of adjacent tip cells, enabling the anastomosis of two independent newly formed vessel sprouts to create a new blood lumen (57). Furthermore, Notch signaling controls the merging properties of tip endothelial cells and stem cells during angiogenesis (58, 59). Macrophage-derived VEGF-C enhances Notch signaling by binding to endothelial cell VEGFR-3 (59). Macrophages also regulate endothelial cell function and angiogenesis by releasing other signaling molecules and factors, such as Sema4D (60), placental growth factor (PlGF) (55), interleukin (IL)-1β, and tumor necrosis factor (TNF)-α (61).

The interaction between vascular endothelial cells and macrophages is bidirectional. Proliferating islet endothelial cells and recruited macrophages cooperate to promote β-cell regeneration (22). In the context of β-cell death, proliferating endothelial cells facilitate the recruitment and phenotypic polarization of macrophages through VEGF-a-VEGFR2 signaling (22). Furthermore, endothelial cells provide a supportive niche for the differentiation and functional polarization of macrophages (62). He et al. developed an *in vitro* co-culture system that facilitated direct interaction between mouse bone marrow-derived hematopoietic cells and a monolayer of liver sinusoidal endothelial cells. This approach led to the formation of macrophage colonies that exhibited characteristics resembling M2-like phenotype. Upon endothelial cell monolayer removal, the structure of the macrophage colonies was disrupted and dissolved.

Among these factors, macrophage colony-stimulating factor (CSF1) is crucial for expanding endothelial-induced macrophage colonies (62). Additionally, the biomarker characteristics of extracellular vesicles (EVs) derived from endothelial and immune cells in metabolic diseases have been well documented (63). Giannella et al. found that miR-126-3p in endothelial cell-derived microparticles from the serum of patients with diabetes was negatively correlated with blood glucose levels (64). Comprehensive research into how extracellular vesicles communicate in macrophages and endothelial cells indicated that the ACO1 protein facilitates the incorporation of miR-503 into small extracellular vesicles (sEVs) originating from M1 macrophages. In a high-glucose environment, these sEVs induce damage and apoptosis in HUVEC endothelial cells by overexpressing miR-503, which targets and inhibits IGF1R expression (65). Infected macrophages also induce endothelial activation and early physiological changes by transporting EVs (66). While some research exists on EVs linked to macrophages from adipose or other tissues, there is insufficient detailed characterization and direct experimental validation concerning EVs and signaling pathways within the islet microenvironment.

### 2.4 Role of intercellular crosstalk among three islet cell types in inflammation and vascular injury in T2DM

Crosstalk among β cells, islet macrophages, and endothelial cells is crucial in T2DM development and progression. Chronic tissue inflammation is a key feature of T2DM. Islet inflammation can cause endothelial injury, which drives islet fibrosis and dysfunction.

**Figure 1:**
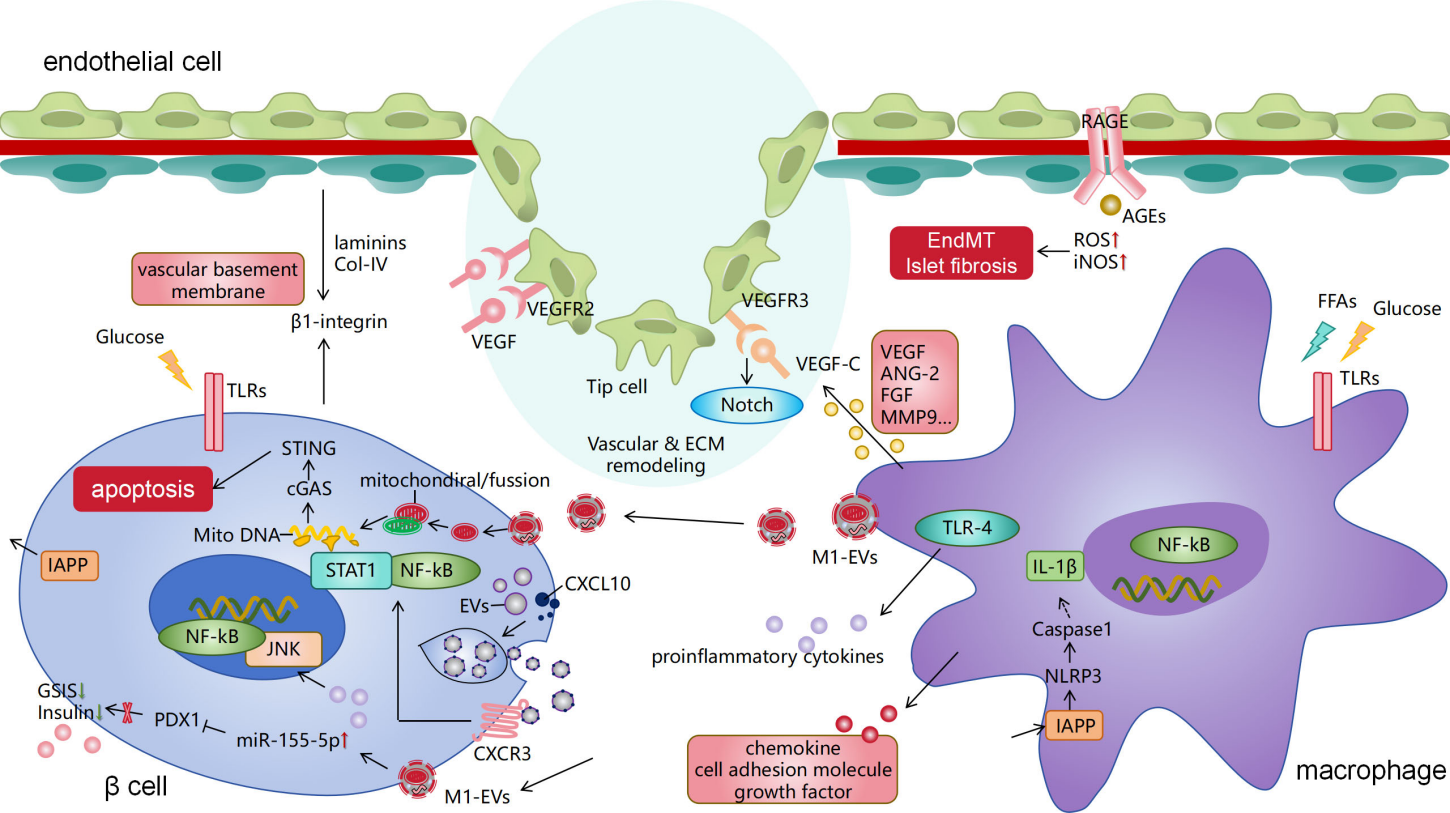
Pathological crosstalk among islet β cells, islet macrophages, and endothelial cells in T2DM. Islet macrophages activate the NLRP3 inflammasome by recognizing IAPP from β cells, leading to the release of IL-1β. They also release other pro-inflammatory factors and chemokines, with the involvement of TLR and NF-κB signaling. This represents a classical pathway of islet inflammation in T2DM. Additionally, islet macrophages communicate with β and endothelial cells by secreting extracellular vesicles (such as exosomes) that deliver RNA cargo and inflammatory mitochondria, damaging both cell types. Islet endothelial cells receive VEGF signals from macrophages, inducing angiogenesis and vascular anastomosis, which is associated with the Notch pathway. Islet ECM components (collagen type I/IV and laminins) also regulate β-cell behavior through integrin signaling. Macrophages also secrete other signaling molecules and factors to regulate endothelial angiogenesis. The binding of advanced glycation end products (AGEs) to their receptor (RAGE) on endothelial cells triggers endothelial-mesenchymal transition, ultimately leading to collagen deposition and fibrosis in T2DM islets. IAPP, islet amyloid polypeptide; T2DM, type 2 diabetes mellitus; IL, interleukin; TLR, Toll-like receptor; NF-κB, nuclear factor kappa B; VEGF, vascular endothelial growth factor.

In this process, the migration, recruitment, and activation of macrophages within the tissue contribute to the formation of chronic low-grade inflammation. β cells are key endocrine cells that reflect the extent of islet dysfunction. The interactions among islet endothelial cells, β cells, and macrophages are vital for islet angiogenesis and repair. The following section outlines the interactions among islet cells in T2DM-related islet inflammation and vascular involvement (**Figure 1**), along with the molecular mechanisms and pathways involved in cell communication (**Table 1**).

**Table 1:** Summary of the mechanistic effects of the crosstalk among islet β cells, islet macrophages, and endothelial cells.

Cell types in the crosstalk	Mediators	Related pathways or mechanisms	Physiological/Pathogenic effects on T2DM	Reference
ECs/MΦs/β cells	Specific overexpression of VEGF-A in induced mouse β cells	VEGF-A signaling pathway and its induced factors in the islet microenvironment promote tissue repair/regeneration	Recruiting macrophages to β-VEGF-A islets; facilitating the formation of the islet microenvironment and the proliferation of β cells following islet damage	(22)
MΦs/β cells	The expression of Pdgfa in islet-infiltrating and perivascular macrophages of HFD mice	PDGF-PDGFR signaling pathway	Promoting β-cell proliferation under obese conditions	(18)
MΦs/β cells	Induced CTGF/CCN2 in a mouse model with 50% β-cell ablation	CTGF	Mediating islet macrophage recruitment and β-cell proliferation following injury	(27)
MΦs/β cells	Upregulation of SMAD7 expression in β cells of a mouse model with pancreatic duct ligation	Activity of SMAD7	Promoting β-cell proliferation by increasing cyclin D1 and cyclin D2 levels and by inducing nuclear exclusion of p27	(28)
MΦs/β cells	Increased expression of IGF-1 in islet macrophages derived from the STZ-induced mouse model	IGF-1 signaling	Contributing to maintaining the insulin secretion level in the second phase of STZ mice; inhibiting pancreatic inflammation	(38)
β cells/MΦs	Upregulated expression of CXCL10 in β cells of pregnant mice	CXCL10-CXCR3 axis	Promoting the accumulation of macrophages in the islets; facilitating the phagocytosis of the physiologically increased β-cell clusters in postpartum mice and restoring glucose homeostasis	(39)
ECs/β cells	Specific overexpression of VEGF-A in β cells of mice	β cell-derived angiogenic factor VEGF-A	Increasing the number of islet ECs but impairing β-cell proliferation and islet morphology	(222)
ECs/β cells	Laminins and other vascular basement membrane proteins (collagen IV and fibronectin)	Constituting the vascular basement membrane together with β1 integrins	Acting as intrinsic signals to promote β-cell proliferation and insulin gene expression	(44)
ECs/β cells	Endothelial-derived-CTGF	CTGF	Autocrine action promoting islet vascular development and paracrine action stimulating β-cell proliferation	(45)
ECs/β cells	Induced CTGF in a mouse model with 50% β-cell ablation	TGF-β signaling	Upregulating positive cell-cycle regulators and factors involved in β-cell proliferation (HGF, 5-HT, and integrin β1), thereby promoting β-cell proliferation and regeneration	(26)
ECs/β cells	Treating T2DM rats with Ang(1-7)	ACE2/Ang(1-7)/Mas pathway	Improving β cell insulin secretion in T2DM rats and reducing islet cell apoptosis; increasing eNOS expression and NO release in the pancreas, dilating pancreatic microvessels, and improving pancreatic microcirculation	(48)
ECs/β cells	Irs2 in mouse ECs	Renin-angiotensin system	Regulating islet blood flow and mediating glucose-induced insulin secretion	(50)
β cells/ECs	The RNA binding protein HuD in β TC6 cells	Acting as a translational repressor to regulate gene expression in islet cells	Regulating the crosstalk between β cells and islet ECs by modulating endostatin and Serpin E1 expression, helping to maintain the balance of the islet microenvironment	(51)
MΦs/ECs	Expression of tissue macrophages and VEGF in gene-mutant mice.	VEGF signaling	Promoting tip cell formation by VEGF gradients and fusing tissue macrophages with these tip cells, facilitating vascular anastomosis	(57)
MΦs/ECs	Vegfr3 in hybrid mouse ECs	VEGF-C–VEGFR-3 signaling and Notch signaling	Macrophage-derived VEGF-C activating VEGFR-3 in endothelial tip cells; aiding in vascular sprout fusion and angiogenesis	(59)
MΦs/ECs	M1 macrophage-derived sEVs miR-503 under high-glucose stimulation	Extracellular vesicle-associated miR-503/IGF1R axis	Impairing EC function and hindering wound healing in patients with diabetes	(65)
MΦs/β cells	High expression of IL-1β in lipopolysaccharide-induced bone marrow-derived macrophages and transgenic mice for human IAPP	Activation of the NLRP3 inflammasome by islet amyloid polypeptide	Acting as an important inflammatory mediator in the pathological mechanisms of T2DM	(71)
MΦs/β cells	The expression of TLR4 in islet macrophages of obese diabetic mice	TLR4-mediated signaling	Altering the islet cytokine secretome, promoting β-cell apoptosis, and potentially being related to inflammation-associated angiogenesis	(78)
β cells/MΦs	Pro-inflammatory β-cell small EVs isolated from inflammatory MIN6 cells	CXCL10/CXCR3 axis, NF-κB and STAT1 activation	Inducing β-cell dysfunction, promoting a pro-inflammatory islet transcriptome, and enhancing recruitment of macrophages	(100)
MΦs/β cells	Macrophage-derived exosomal miR-155 isolated from bone marrow-derived macrophages of diabetic mice	MiR-155-PDX1 axis	Mediating islet inflammation and β-cell dysfunction; promoting the progression of T2DM	(101)
MΦs/β cells	Release of EVs from M1 macrophages in mouse islets and inflammatory mitochondria	The ferroptosis pathway, mitochondrial disruption, and activation of the STING pathway	Resulting in lipid peroxidation and mitochondrial disruption and inducing ferroptosis in pancreatic β cells	(102)
MΦs/β cells	Expression of STING in palmitic acid-treated mouse bone marrow-derived macrophages	cGAS-STING pathway	Impairing glucose-stimulated insulin secretion by mediating the engulfment of β cell insulin secretory granules	(104)
β cells/MΦs	Expression of miR-29 in islet-derived exosomes from HFD-fed mice	MiRNA-29-TNF-receptor-associated factor 3 (TRAF3) axis	Promoting the recruitment and activation of circulating monocytes and macrophages; driving macrophage-dependent systemic inflammation; promoting the occurrence of inflammation and diabetes	(107)
Ecs/β cells	Increased expression of AGEs in islet ECs of diabetic mice	AGE-induced islet EndMT	Inducing EndMT in islet ECs and islet fibrosis in diabetic mice	(91)

VEGF-A, vascular endothelial growth factor A; MΦs, macrophages; HFD, high-fat diet; PDGF, platelet-derived growth factor; CTGF, connective tissue growth factor; IGF-1, insulin-like growth factor-1; ECs, endothelial cells; eNOS, endothelial nitric oxide synthase; NO, nitric oxide; T2DM, type 2 diabetes mellitus; sVEs, small extracellular vesicles; IAPP, islet amyloid polypeptide; EVs, extracellular vesicles; TLR, Toll-like receptor; EndMT, endothelial-to-mesenchymal transition; AGEs, advanced glycation end products.

The listed cell-type pairs indicate experimentally observed functional interactions; these communications are likely bidirectional and context-dependent.

#### 2.4.1 Macrophage infiltration and inflammation

In the early stages of T2DM, the infiltration of macrophages in the islets leads to the secretion of various inflammatory factors, triggering islet inflammation and impairing β-cell function and insulin secretion (16, 67). This infiltration is primarily characterized by the accumulation of M1 macrophages (68), which may result from the local proliferation of resident islet macrophages (18). Increased secretion of inflammatory factors, such as IL-6, IL-8, chemokine KC, and macrophage inflammatory protein 1 alpha, from T2DM islets positively correlates with the number of islet-associated macrophages (69). Among these, IL-1β is a significant inflammatory mediator in the crosstalk between islet macrophages and β cells in T2DM (70). The NLRP3 inflammasome induces islet macrophages to release IL-1β by recognizing IAPP and other T2DM-associated metabolites (71), further promoting β-cell damage and T2DM progression.

Toll-like receptors (TLRs), pattern recognition receptors, can identify pathogens and sense damage-associated molecular patterns (DAMPs) from damaged cells, thereby initiating inflammatory signaling cascades. TLRs are highly expressed in animal models of diabetic insulin resistance (72, 73). High glucose and free fatty acids stimulate TLRs, activating downstream inflammatory pathways and expression of inflammatory factors (74, 75). Enhanced TLR4 signaling in diabetic islets can exacerbate inflammatory responses (70, 76, 77). Recent studies have demonstrated that 90% of TLR4^+^ signaling in diabetic islets originates from islet macrophages and endothelial cells, with islet macrophages being the primary source (78). In obese T2DM mice, islet macrophages promoted increased secretion of pro-inflammatory cytokines mediated by TLR4. Importantly, the heightened immune response of TLRs (TLRa2-4^+^) in diabetic islets is closely associated with the proliferation of endothelial cells and macrophages (78).

In addition to the above, islet inflammation is a key pathological process that involves multiple other signaling pathways. For instance, the NF-κB signaling pathway is crucial in regulating inflammatory responses (79). It regulates the transcription of genes such as cytokines, chemokines, and adhesion molecules, essential for modulating islet inflammation (80). Evidence suggests that the NF-κB signaling pathway plays a role in islet inflammation (81), including the phosphorylation of SER536 and the nuclear translocation of p65, representing the classic pathway of NF-κB activation (82, 83). In the context of aging-associated chronic inflammation in zebrafish islets, macrophage recruitment and partial expression of the cytokine TNFα are observed. β-cells upregulate the TNFα receptor *TNFRSF1B*, thereby triggering NF-κB activation (84). Interestingly, β-cells in aged fish with higher NF-κB activity express higher levels of *socs2*, an age-related gene that inhibits β-cell proliferation. Thus, the heterogeneity of NF-κB signaling in β-cells may be associated with gene expression and proliferation.Additionally, the activation of the JAK-STAT pathway is closely related to the production and action of various inflammatory factors. When cytokines (such as IFN-γ and IL-6) bind to their receptors, the JAK proteins associated with the receptors are activated. Activation of JAK proteins further leads to the phosphorylation and dimerization of STAT proteins, followed by the translocation of STAT dimers to the nucleus, where they bind to specific *DNA* sites and regulate gene transcription (85, 86). This process is critical in islet inflammation, as it can promote the production and release of inflammatory factors, exacerbating the inflammatory response. Recent studies have shown that the *Apolipoprotein L (APOL)* gene is a novel regulator of islet inflammation, expressed in human pancreatic β-cells (87). Under T2DM islet inflammatory conditions, the JAK-STAT pathway mediates the upregulation of *APOL1, APOL2, and APOL6* expression. Moreover, *mammalian target of rapamycin (mTOR)* is a key nutrient-sensitive signaling hub that controls β-cell metabolism and function (88). β-cells from patients with T2DM and mouse models exhibit hyperactivation of *mTORC1* and hypoactivation of *mTORC2* (89). Sustained *mTORC1* activity impacts islet inflammation in T2DM, thereby promoting β-cell dysfunction (88). Studies have shown that mTOR can coordinate with downstream signaling to alleviate macrophage inflammation, thereby preventing β-cell dysfunction. For example, by regulating the mTOR/S6/4E-BP1 signaling pathway, reversing the downregulation of *PDX1*, and inhibiting the upregulation of *ALDH1A3* in β-cells, it is possible to suppress islet macrophage accumulation and M1-like polarization in obese mice, improving β-cell function (90).

#### 2.4.2 Islet angiogenesis and vascular remodeling

To accommodate the expansion of β-cell clusters early in the disease, there is a compensatory increase in the perfusion of the islet vascular system, accompanied by endothelial cell proliferation (42, 91). During this period, islet macrophages are crucial in promoting islet angiogenesis, remodeling, and insulin secretion. The number of islet macrophages also increases in response to the pathological changes in the islets. In fact, the absence of islet macrophages reduces the secretion of VEGF-A by the islets, hindering vascular remodeling, and impairs islet function and morphology.

When islets with macrophage depletion were transplanted into high-fat-fed db/db mice, islet vascular reconstruction was delayed; however, supplementing VEGF-A improved the weakened islet vascularization. This suggests that islet macrophages contribute to the formation of a pro-angiogenic islet environment during early diabetes, promoting islet remodeling, compensatory hyperinsulinemia, and controlling diabetes. As T2DM progresses, islet capillaries gradually thicken, dilate, and break, accompanied by islet endothelial cells showing inflammatory and advanced glycation end product (AGE) markers (92). In this process, endothelial dysfunction may occur through the abnormal downregulation or upregulation of beneficial factors or mediators discussed above (HGF, CTGF, THBS1, and laminins), leading to impaired insulin release from β cells. Therefore, islet endothelial cells are a novel factor in β-cell dysfunction in T2DM (46). Moreover, in the *RIP1-Tag2* pancreatic islet carcinoma model, *angiopoietin-2 (ANG2)* has been found to regulate the biological characteristics of endothelial cells by binding to *TIE2* expressed by tumor-associated macrophages (TEMs), thereby promoting angiogenesis and vascular remodeling (93). Targeting the ANG2/TIE2 signaling pathway may be crucial in pancreatic tumor-associated angiogenesis.

As noted above, endothelial-derived laminins and collagen IV enhance β-cell proliferation and function. Specific ECM configurations—such as collagen IV combined with laminin-derived RGD, LRE, or PDSGR motifs—restore insulin-secretory pathways and suppress inflammatory and hypoxic responses when incorporated into alginate microcapsules (94, 95). Pancreatic collagens I and IV not only provide mechanical scaffolding for islets but also engage cell-surface receptors to modulate β-cell behavior (96). Integrin α3β1 binding to collagens I/IV orchestrates β-cell survival and function through FAK–ERK1/2 and PI3K/Akt cascades (97), whereas collagens I and V, via an integrin-β1/E-cadherin/β-catenin axis, specifically drive proliferation of rat INS-1 β-cells (98). *De novo* assembly of a peri-islet basement membrane on ECM-deficient stem cell-derived beta-like cells further demonstrates that precisely engineered ECM–cell interactions constitute a robust strategy for the ex vivo expansion and transplantation of functional β cells (99).

#### 2.4.3 Role of EVs in β-cell damage

EVs originating from the pancreas contribute to β-cell damage (63). Under the stress conditions of T2DM, β cell-derived EVs mediate the activation of the islet CXCL10/CXCR3 axis (100). Specifically, these EVs are enriched with the chemokine CXCL10, which binds to CXCL10 receptors on β-cell surface, leading to the transcriptional activation of downstream pro-inflammatory pathways NF-κB and STAT1, further exacerbating the expression of inflammatory factors and CXCL10 (100). The pro-inflammatory β-cell EVs also promote the recruitment of immune cells, such as macrophages and T cells, to the islets through the CXCL10/CXCR3 pathway. Moreover, macrophages promote islet inflammation by transferring miR-155 and inflammatory mitochondria to β cells through EVs (101, 102). MiR-155, derived from M1 macrophages in the islets, enters β cells via exosomes and targets PDX1, a characteristic regulator of β-cell function, thereby contributing to β-cell damage and T2DM progression of T2DM (101).

PDX1 is also associated with increased DNA methylation in islets of patients with T2DM (103), suggesting that communication between islet cells is linked to the epigenetics of T2DM. Additionally, EVs from M1 islet macrophages can transport inflammatory mitochondria into β cells, where they fuse with the mitochondria, inducing lipid peroxidation and mitochondrial fragmentation, which leads to the release of mitochondrial DNA into the cytoplasm. This activates the STING pathway, triggering β-cell death (102). The phagocytic activity of islet macrophages and the STING pathway have attracted attention. Similar mechanisms indicate that in high-fat diet models, the release of mitochondrial DNA into the cytoplasm activates cGAS-STING signaling, mediating the phagocytosis of insulin granules by islet macrophages (104). Thus, activating the STING signaling pathway in pathological states can enhance the interaction between islet macrophages and β cells, impacting insulin secretion. Although STING is highly expressed in both mouse and human β cells (105, 106), its expression and role in macrophages in T2DM patients with obesity require further investigation. Furthermore, β cells can promote macrophage activation and recruitment in a TNF-receptor-associated factor 3 (TRAF3)-dependent manner through miR-29 exosomes and the expression of miR-29, accelerating the onset of diabetes and inflammation (107).

#### 2.4.4 Advanced glycation end products and vascular injury

In the later stages of T2DM, accelerated advanced glycation end products (AGEs) formation occurs (108). AGEs are typically found in the vascular walls (109, 110) and can bind to their receptor (RAGE) expressed on endothelial cells, triggering harmful pro-inflammatory responses (111). Monocytes and macrophages express AGE receptors similar to those on endothelial cells. The role of AGE-ligand-receptor interactions in the pathological development of diabetes-related vascular tissues has been extensively reviewed (108). In diabetic mice subjected to femoral artery ligation to induce ischemic peripheral vascular disease, studies have shown that angiogenesis and blood flow recovery are impaired. This impairment is accompanied by a reduced macrophage content in ischemic muscle and suppressed expression of early inflammatory genes (*Ccl2* and *Egr1*) (112). Furthermore, interactions between macrophages and endothelial cells cultured *in vitro* under high-glucose conditions are also diminished. Antagonism of the AGE/RAGE axis can improve ischemia and angiogenesis in the diabetic peripheral vasculature, restoring adaptive inflammation in macrophages within the inflammatory microenvironment.

The AGE/RAGE interaction can stimulate the production of ROS and induce the expression of inducible nitric oxide synthase (iNOS) (113). Recent studies have detected glycation end products in aging islet vasculature, while they are absent in the islet cells themselves. Additionally, increased iNOS levels have been reported in the islet vasculature of C57BL/6J mice corresponding to sites of AGE accumulation (114). Further investigation revealed that AGE accumulation in the islets of diabetic mice triggers an endothelial-mesenchymal transition in the islet endothelial cells (characterized by increased α-SMA and fibronectin expression and decreased CD31 and VE-cadherin expression), leading to collagen deposition and fibrosis in the islets (115). This represents a significant pathophysiological mechanism for the progressive decline and ultimate failure of β-cell function (116, 117). Changes in islet vascular fibrosis also exacerbate the progression of diabetes and its complications (118).

## 3 Natural products for management of T2DM by modulating cellular crosstalk in the pancreatic microenvironment

### 3.1 Berberine

Berberine is an alkaloid isolated from various plants, such as *Coptis chinensis* (119). In the context of pancreatic inflammation, berberine reduces the levels of cytokines and chemokines such as IL-1β, IFN-γ, TNF-α, and MCP-1 through various pathways, improving tissue inflammation and protecting pancreatic cells (119, 120). Berberine has been shown to protect STZ-treated mouse primary pancreatic cells from apoptosis by downregulating the ratio of *Bax/Bcl-2* apoptosis genes in islets (121). Wang et al. have shown that berberine alleviates lipopolysaccharide (LPS)-induced β-cell inflammation and apoptosis by blocking TLR4 signaling and the downstream JNK/NF-κB pathway (122), which is closely related to the inflammation and β-cell dysfunction in T2DM (122). In addition, berberine activates AMP-activated protein kinase (AMPK), positively regulating pancreatic β-cell function (123) and alleviating macrophage pro-inflammatory responses (124). Berberine activates AMPK in pancreatic β cells (125) and regulates insulin gene transcription by inhibiting the expression of mouse insulin promoter, mRNA, and protein through the AMPK pathway, thereby playing a therapeutic role in T2DM with hyperinsulinemia (123). In LPS-induced mouse primary peritoneal macrophages, berberine significantly inhibited the expression of IL-1β, IL-6, iNOS, MCP-1, and COX-2 (124). Berberine also suppressed the phosphorylation of MAPKs, such as p38, ERK, and JNK, in macrophages under inflammatory stimulation, as well as ROS and NO levels. The inhibitory effect of berberine on these pro-inflammatory responses involves the activation of AMPK (124). When taken orally, berberine is converted in the intestine into an important oxidative metabolite, oxidized berberine (OBB), which is absorbed into the bloodstream, binds to hemoglobin, and is taken up (126) and released by macrophages. OBB alleviates systemic inflammation levels and β-cell oxidative stress by inducing HO-1 protein expression and activating the PI3K/AKT and AMPK pathways, thereby improving β-cell function and insulin secretion (127) (**Table 2**). Multiple clinical randomized controlled trials have demonstrated that berberine significantly improves fasting blood glucose, glycated hemoglobin, lipid profiles, and other metabolic markers in patients with diabetes (128–130). These findings support the incorporation of berberine into management strategies for T2DM.

**Table 2:** Treatment of T2DM through the regulation of islet-cell crosstalk by herbal monomers.

Classification	Models		Pathways	Relevant genes/proteins (↓ down-regulation; ↑ up-regulation)	Outcome	Reference
	*In vivo*	*In vitro*				
Berberine	–	Primary islet cell model from ICR mice treated with STZ	Bax/Bcl-2 (pro-/anti-apoptotic) gene expression	↓: Bax/Bcl-2 gene expression ratio	Inhibiting islet-cell apoptosis	(121)
Berberine	–	LPS-induced injury model in NIT-1 cells and rat insulinoma (INS-1) cells	TLR4-independent JNK/NF-κB pathway	↓: MCP-1, IL-6, and TNF-α; insulin, JNK, and NF-κB phosphorylation in NIT-1 cells; p65 NF-κB in INS-1 cells	Improving LPS-induced β-cell injury	(122)
Berberine	Rat model fed an HFD	The MIN6 cell model induced by palmitic acid and cAMP elevating agents	AMPK signaling pathway	↑: Phosphorylation of AMPK and ACC ↓: PKA activity	Significantly inhibiting glucose-stimulated insulin secretion in MIN6 cells and rat islets	(125)
Berberine	HFD-induced C57BL/6J mouse model	NIT-1 cell model induced by different concentrations of glucose medium	AMPK signaling pathway	↑: Phosphorylating AMPK and its downstream molecule acetyl-CoA carboxylase (ACC) ↓: Ins2 mRNA, insulin protein, and MIP2 activity	Improving insulin resistance, impaired glucose tolerance, and hyperinsulinemia in obese mice; inhibiting the activity of the mouse insulin gene promoter to slow down β-cell metabolism	(123)
Berberine	–	LPS-induced RAW 264.7 cell and primary mouse peritoneal macrophage models	AMPK signaling pathway	↓: IL-1β, IL-6, iNOS, MCP-1, COX-2, MMP-9, and ROS; phosphorylation of MAPKs (p38, ERK, and JNK)	Inhibiting the pro-inflammatory response of macrophages	(124)
Quercetin	–	Tunicamycin-induced human umbilical vein endothelial cell model	Endoplasmic reticulum stress	↑: Bcl-2, SOD1, and catalase ↓: MDA, Bax, GRP78, CHOP, and caspase 3	Alleviating endoplasmic reticulum stress in endothelial cells and protecting endothelial function	(132)
Quercetin	STZ-induced diabetic Wistar rat model	–	Endoplasmic reticulum stress	↑: VEGF, VEGFR2, SOD, CAT, GPx, and GSH ↓: ET-1, CHOP, nitrite, cGMP, MDA, and LPO	Alleviating endoplasmic reticulum stress damage in the pancreatic vasculature of diabetic rats, improving pancreatic endothelial function, and enhancing the morphology and quality of β cells	(133)
Quercetin	–	IL-1β-induced rat insulinoma cell line RINm5F model	NF-κB signaling pathway	↓: iNOS and iNOS promoter activity; IL-1β-induced activation of NF-κB binding activity; IκBα phosphorylation; nuclear translocation of p65	Inhibiting the release of the cytokine IL-1β from pancreatic inflammatory cells, along with its induced NF-κB activation and iNOS gene expression, and restoring suppressed insulin secretion	(137)
Quercetin	STZ-induced diabetic Wistar rat model	–	Oxidative stress	↑: SOD, GSH-Px, and CAT ↓: MDA and NO	Alleviating pancreatic oxidative stress and β-cell damage in diabetic rats and protecting β-cell integrity	(131)
Quercetin	–	Human amylin (8–37) induced Rat insulinoma pancreatic cell (RIN-m5F) model	HIAPP	↑: Viability of amylin-challenged RIN-m5F cells	Inhibiting the aggregation of amylin *in vitro* in pancreatic cells to protect cells against the cytotoxic effects of amylin	(140)
Astragalus polysaccharide	–	Macrophage model derived from THP-1 monocytes	Binding of S proteins to recombinant ACE2	↑: IL-10, IL-1RN, CD163, and CD206	Promoting the polarization of THP-1-derived macrophages to the M2 anti-inflammatory phenotype	(144)
Astragalus polysaccharide	STZ-induced diabetic ulcer model in rats	–	β-catenin/NF-κB axis	↑: β-catenin and Rspo3 ↓: NF-KB and GSK-3β	Promoting macrophage polarization to the M2 type to alleviate excessive inflammatory responses in wound healing	(145)
Astragalus polysaccharide	–	Palmitate-induced RAW 264.7 cell inflammation model	AMPK activity	↑: IL-10, MMR, Dectin-1, arginase, YM-1, and YM-2 ↓: IL-1β, iNOS, MCP-1, IL-6, and CD11c	Improving the pro-inflammatory response of macrophages, which may be associated with the mitigation of diabetes and insulin sensitivity *in vivo*	(149)
*Astragalus membranaceus* (APS-A1 and APS-B1)	–	LPS-induced inflammatory RAW 264.7 mouse macrophage model	NF-κB and MAPK (ERK and JNK) pathways	↓: TNF-α, IL-1β, IL-6, and MCP-1; NLRP3, iNOS, and COX-2; phosphorylation of MAPK; NF-κB activation and P65 nuclear translocation	Inhibiting the secretion of inflammatory mediators to reduce the inflammatory response, which may be related to their structure-function relationship	(150)
Curcumin	T2DM rat model induced by an HFD and STZ intraperitoneal injection	–	RAGE/JNK/NF-κB signaling pathway	↑: Bcl-2, superoxide dismutase 2, and glutathione peroxidase ↓: IL-1β, IL-6, TNF-α, caspase 3, Bax, and malondialdehyde; phosphorylated JNK and NF-κB proteins	Inhibiting inflammation and apoptosis in pancreatic islet β cells	(153)
Curcumin	HFD-induced C57BL/6J diabetic mouse model	–	NF-κB signaling pathway	↓: Hepatic TNF-α, SOCS-3, MCP-1, and chemokine ligand-2; p65 activity and NF-κB activity ↑: adipose tissue Foxo1	Reducing the infiltration of macrophages in white adipose tissue, reversing obesity-related inflammation and metabolic disorders, and improving blood glucose levels in diabetic mice	(156)
Curcumin	–	β-Min6 cell model cultured in high and low glucose	AMP-dependent signaling pathway	↑: cAMP ↓: the 11 PDE isozymes, including PDE3B, PDE8A, and PDE10A; PDE activity	Enhancing pancreatic β-cell function	(157)
Curcumin mono-carbonyl analogues C66	Primary peritoneal macrophage model from high glucose-treated C57BL/6 mice	–	JNK/NF-kB signaling pathway	↓: TNF-α, NO, IL-1β, IL-6, IL-12, COX-2, and iNOS mRNA transcription	Inhibiting renal inflammation in diabetic mice, reducing macrophage infiltration in the renal interstitium, and improving renal histological abnormalities and fibrosis	(158)
Novel chemically-modified curcumin 2.24	–	Inflammatory macrophage model established by treating SD rat peritoneal macrophages with LPS/AGE	Changing cell phenotype	↑: RvD1 and sRAGE secretion ↓: IL-1β, IL-6, and MMP-9	Promoting the polarization of macrophages from M1 to M2 phenotype to reduce inflammation	(159)
Luteolin	–	IL-1β- and IFN-γ-induced RINm5F rat insulinoma cell damage model	NF-κB activation	↓: IL-1β- and IFN-γ-induced NO production, NF-κB binding activity	Inhibiting cytotoxicity in RIN cells, improving insulin secretion, and alleviating damage to pancreatic β cells	(164)
Luteolin	–	Palmitate (PA)-induced INS-1E (rat insulinoma) cell apoptosis model	Regulation of autophagy and ROS clearance	↓: Apoptosis marker proteins PARP, cleaved caspase 3, and cleaved caspase 9; ROS; Drak2 activity	Improving islet β-cell dysfunction via autophagy promotion and its antioxidative effect	(165)
Luteolin	HFD- and STZ-induced T2DM rat model	–	PPAR-γ, SREBP-1c, and NF-κB signaling pathways	↑: PPAR-γ expression ↓: TNF-α, IL-6, and NF-κB levels; SREBP-1c expression	Alleviating inflammation and dysregulation of cytokine secretion, improving hyperglycemia and low insulin levels, β-cell dysfunction, and kidney damage in diabetic rats	(166)
Luteolin	HFD-fed ovariectomized C57BL/6J mouse model	Primary bone marrow-derived macrophage model from mice and J774A.1 macrophage model	NLRP3 inflammasome	↓: mRNA expression of NLRP3 inflammasome components ASC and caspase 1; IL-1β and TNF-α	Protecting against inflammatory diseases by inhibiting the induction and activation of NLRP3	(168)
Luteolin	HFD-fed obese C57BL/6 mouse model	LPS- and PIG-stimulated mouse RAW264.7 cells and C57BL/6 mouse peritoneal macrophage model	AMPKα1 signaling pathway	↑: Levels of the M2 marker Arg1 ↓: MMe markers Cd36 and Plin2 in EATs; pro-inflammatory cytokine genes Mcp1, TNF-α, and IL-6; expression of the M1 marker Nos2; Akt phosphorylation	Inhibiting inflammatory macrophage polarization and reducing obesity-related insulin resistance	(169)
Puerarin	HFD-induced diabetic mouse model	Isolated cultured mouse pancreatic ductal cells	GLP-1R/Wnt/STAT3 signal transduction	↑: Transcription factor PDX-1; GLP-1R; transcription factor neurogenin 3 (Ngn3); islet-like cell clusters; activity of β-catenin and STAT3	Improving glucose homeostasis in diabetic mice and promoting β-cell neogenesis	(171)
Puerarin	–	H_2_O_2_-treated MIN6 cell model	PERK-eIF2-ATF4-CHOP pathway and JAK2/STAT3 signaling	↑: Anti-apoptotic proteins (Bcl-2 and MCL1) ↓: Pro-apoptotic protein (Bax); cleaved caspase 9/12 and cleaved caspase 3/7; GRP78; phosphorylated PERK; phosphorylated eIF2α; ATF4; CHOP	Attenuating endoplasmic reticulum stress and preventing MIN6 cells from apoptosis	(172)
Puerarin	STZ-induced diabetic Wistar rat model	–	TGF-β1/Smad2 pathway	↑: Levels of IL-4, SOD, CAT, GSH-Px, and NO ↓: Levels of MDA, IFN-γ, and IFN-γ/IL-4; mRNA and protein expression of TGF-β1, Smad2, CTGF, and FN	Exerting anti-diabetic effects; improving renal function, which may be related to its antioxidant properties	(173)
Puerarin	HFD combined with STZ injection induced T2DM mouse model	–	Caspase/AIF/apoptosis pathway	↓: caspases 3, 8, and 9 and AIF proteins	Protecting β cells from apoptosis	(174)
Puerarin	STZ injection-induced diabetic C57BL/6 mouse model	CoCl2-induced mouse insulinoma MIN6 cell apoptosis model	PI3K/Akt signaling pathway	↑: Bcl2/Bax; AKT phosphorylation ↓: ROS generation	Protecting pancreatic β-cell function and survival	(175)
Puerarin	–	H_2_O_2_-induced mouse insulinoma MIN6 cell apoptosis model	Cellular oxidative stress	↓: Intracellular ROS; cleaved caspase 3	Alleviating oxidative stress levels in β cells, protecting mitochondria, and promoting β-cell survival	(176)
Puerarin	STZ-induced diabetic C57BL/6 mouse model	LPS-induced RAW264.7 cell inflammation model	NF-κB and MAPK signaling cascades	↑: Arg-1, IL-10, and TGF-β1 ↓: p-STAT3, TNF-α, IL-1β, IL-6, p65, IkBα, ERK, JNK, and p38	Reducing tissue macrophage infiltration, inducing polarization of M2 macrophages, and inhibiting the activation of inflammatory pathways	(177)
Puerarin	–	High concentration FFA-induced RAW264.7 macrophage inflammation model	P2X4R signaling	↓: TNF-α, iNOS, NO, and P2X4R	Alleviating the inflammatory response of macrophages induced by high concentration FFA	(178)

LPS, lipopolysaccharide; NO, nitric oxide; T2DM, type 2 diabetes mellitus; IL, interleukin; iNOS, inducible nitric oxide synthase; HFD, high-fat diet; NF-κB, nuclear factor kappa B; PDE, phosphodiesterase; MMP, metalloproteinase; hIAPP, human islet amyloid polypeptide; ROS, reactive oxygen species; VEGF, vascular endothelial growth factor; AGE, advanced glycation end product; AIF, apoptosis-inducing factor; FFAs, free fatty acids.

### 3.2 Quercetin

Quercetin, a beneficial flavonoid natural product found in various vegetables and fruits (131), improves diabetes-induced endothelial dysfunction and pancreatic islet injury. The former primarily occurs by reducing endothelial cell apoptosis (132), alleviating endoplasmic reticulum stress in the pancreas, and increasing the expression of VEGF and its receptor VEGFR2 (133). Notably, VEGF-B is expressed in pancreatic β cells and signals to vascular endothelial cells (134). Inhibition or reduction of VEGF-B signaling can regulate lipid transport in the islet endothelial cells of diabetic mice, mitigate NLRP3 inflammasome activation (135), and decrease cleaved caspase 1-mediated IL-1β expression (136), thereby restoring insulin sensitivity and protecting pancreatic β cells. Studies have compared the inhibitory effects of quercetin and its metabolites on IL-1β and found that quercetin significantly inhibits IL-1β-induced NF-κB activation and iNOS activity, aiding in restoring insulin secretion; however, its metabolites do not exhibit this effect (137). Additionally, quercetin reduces lipid peroxidation, NO and cGMP levels, and antioxidant enzymes in diabetic rats, improving oxidative stress and protecting the pancreatic vasculature (131). Targeting pancreatic IAPP, an important mediator of quercetin’s influence on the crosstalk between β cells and macrophages, with quercetin is considered a novel mechanism for treating T2DM (138). Molecular dynamic simulations indicate that the aromatic properties of quercetin are key factors in inhibiting IAPP (20-29) aggregation (139). Quercetin competitively interacts with the aromatic residues in IAPP through its aromatic ring, preventing “π-π stacking” of IAPP and inhibiting the interactions between peptide Phe23 and the trimerization of IAPP (20-29), thus preventing protein misfolding, aggregation, and amyloid formation in pancreatic β cells (138, 140).

### 3.3 Astragalus polysaccharides

Astragalus polysaccharides, a mixture of polysaccharides extracted from *Astragalus membranaceus* (141), have been shown to reduce β-cell apoptosis and protect against immune damage by modulating immune cell functions and cytokine levels (142). Promoting the polarization of macrophages toward an M2 anti-inflammatory phenotype helps alleviate diabetic inflammation and improve β-cell function (143). Treating THP-1-derived macrophages with Astragalus polysaccharides can increase CD163 expression, facilitating the conversion of macrophages to the M2 phenotype (144). In a diabetic rat ulcer model, Astragalus polysaccharides stimulated macrophage polarization toward M2 by inhibiting GSK-3β, promoting β-catenin expression, and suppressing the NF-κB inflammatory pathway, thereby reducing the inflammatory response (145). In islet macrophages of high-fat-fed diabetic mice, targeting the downregulation of inflammatory M1 macrophage-derived exosomal miR-212-5p can improve β-cell dysfunction and insulin secretion (146). Mechanistically, exosomal miR-212-5p impairs insulin functionality in β cells by targeting its downstream target SIRT2 and regulating the Akt/GSK-3β/β-catenin pathway. This suggests a potential link between the regulation of the GSK-3β/β-catenin pathway by Astragalus polysaccharides and the crosstalk between islet macrophages and β cells. Notably, Astragalus polysaccharides act as ligands for TLR4 due to their unique structure (147) and can induce the activation of NF-κB and the release of cytokines in RAW264.7 macrophages through the phosphorylation of TLR4-related MAPK pathways (148) Evidence supports the role of Astragalus polysaccharides in mitigating diabetes and exhibiting anti-inflammatory effects *in vivo* and *in vitro*. For example, Astragalus polysaccharides reduce palmitate-induced pro-inflammatory responses in macrophages through AMPK activation (149). Chen et al. isolated two new polysaccharides (APS-A1 and APS-B1) from Astragalus polysaccharides and demonstrated their ability to diminish LPS-induced MAPK phosphorylation in RAW264.7 macrophages, inhibiting NF-κB activation and p65 nuclear translocation while reducing the production of TNF-α, IL-6, and MCP-1, thereby exerting anti-inflammatory effects (150). Additionally, other isoflavones and saponins isolated from the Astragalus root have been shown to inhibit NF-κB activation and the release of inflammatory factors in macrophages via the MAPK pathway (151).

### 3.4 Curcumin

Curcumin is a polyphenolic compound extracted from the rhizome of the herb turmeric, known for its antioxidant, anti-inflammatory, and potential benefits against obesity and diabetes (152). Curcumin can specifically inhibit the JNK and NF-κB inflammatory signaling pathways in various cells, including pancreatic β cells and macrophages, which are closely related to T2DM pathogenesis of T2DM (153–155). One study indicated that treatment with high-dose oral curcumin in high-fat diet-induced C57BL/6J obese diabetic mice reduced NF-κB activity and macrophage infiltration in the liver and adipose tissue, thereby improving blood glucose levels and insulin sensitivity (156). Curcumin also mediates the protection of β cells from oxidative stress and cytokine-induced apoptosis by activating Nrf2, inducing HO-1 expression, increasing pancreatic glutathione and antioxidant enzymes, and scavenging free radicals (155). In a T2DM rat model, curcumin alleviated the expression of pro-apoptotic proteins caspase 3 and Bax while increasing the expression of antioxidant and anti-apoptotic proteins such as GSH-PX, SOD2, and Bcl-2 (153). Additionally, in human pancreatic β-cell line HP62 and mouse β-Min6 cells, curcumin inhibited the expression of phosphodiesterase (PDE), an enzyme that hydrolyzes cAMP in islets and β cells, leading to increased intracellular cAMP levels and enhanced insulin secretion, thereby improving β-cell function (157). Pan et al. discovered C66, a novel curcumin derivative that overcomes the challenge of low bioavailability and exhibits strong anti-inflammatory activity. C66 improves the inflammatory response in high-glucose-induced primary peritoneal macrophages from mice, primarily by inhibiting JNK/NF-κB signaling activation (158).

C66 treatment also reduces macrophage infiltration in the renal interstitium of diabetic rats and decreases glomerular microvascular sclerosis, accompanied by reduced expression of MCP-1 and TNF-α (158). Moreover, a new chemically modified curcumin compound, CMC2.24, improved anti-inflammatory effects by regulating macrophage polarization. Flow cytometry showed a significant increase in the M2/M1 ratio of monocytes/macrophages in the CMC2.24 treatment group compared with that in untreated and model groups, indicating a shift in macrophage phenotype from M1 to M2 (159). Similar novel compounds with anti-inflammatory properties may have therapeutic effects against chronic inflammatory diseases, including diabetes and inflammatory organs, through inflammatory mechanisms. In a 12-month randomized controlled trial, curcumin extract significantly reduced blood glucose levels and improved overall β-cell function in obese patients with T2DM (160). Other clinical studies have also shown that curcumin has beneficial effects in reducing the incidence of diabetic complications (161, 162). These studies further confirm curcumin’s efficacy and therapeutic potential in managing T2DM.

### 3.5 Luteolin

Luteolin is a flavonoid polyphenolic compound found in herbs, fruits, and vegetables (163), known for its antidiabetic, anti-inflammatory, and antioxidant pharmacological benefits. Its protective effects on pancreatic β cells include promoting autophagy, alleviating inflammatory responses, and reducing oxidative stress. Luteolin protects β cells from NO and NF-κB-induced cytotoxicity by lowering IL-1β and IFN-γ levels, thereby promoting insulin secretion (164). Luteolin also promotes autophagy by targeting and inhibiting the activity of the apoptosis-promoting kinase Drak2, thus protecting β cells from apoptosis. Additionally, luteolin reduces ROS production and oxidative stress in rat insulinoma INS-1 cells, contributing to the reduction of Drak2 (165). Shehnaz et al. found that luteolin improved β-cell function and high insulin status in the pancreas of high-fat diet and STZ-induced T2DM rats, primarily related to increased PPAR-γ expression and decreased SREBP-1c expression, along with a reduction in inflammatory mediators such as TNF-α, IL-6, and NF-κB (166). Luteolin also exerts significant anti-inflammatory effects by targeting macrophages. A prospective cohort study indicated that the reduction in all-cause mortality among patients with T2DM associated with luteolin may relate to its anti-inflammatory properties (167).Lee et al. showed that luteolin could inhibit NLRP3 inflammasome activation in primary macrophages from high-fat diet mice, suppressing caspase 1 and IL-1β expression, possibly by inhibiting ASC oligomerization (168). Whether this is related to the hydrolytic effects of luteolin on ATPases remains to be determined. *In vivo* studies of mouse peritoneal macrophages and *in vitro* studies using RAW264.7 macrophages revealed that luteolin could directly inhibit M1 inflammatory polarization in macrophages by activating AMPKα1 signaling, thereby improving insulin resistance and tissue inflammation in high-fat diet-induced mice (169). Overall, these findings confirm that luteolin interacts through multiple mechanisms and complex patterns of intercellular and intracellular signaling, directly or indirectly regulating the functions of pancreatic β cells and macrophages, thus contributing to the treatment of diabetes.

### 3.6 Puerarin

Puerarin is an isoflavone monomer extracted from the dried roots of the leguminous plant *Pueraria lobata*, known for its pharmacological activities, including regulating glucose and lipid metabolism, alleviating insulin resistance, and exhibiting antioxidant and anti-inflammatory effects (170). Puerarin promotes the conversion of pancreatic ductal cells into β cells by activating the GLP-1 pathway in high-fat diet fed diabetic mice and upregulating β-catenin and STAT3 levels, thereby facilitating β-cell neogenesis.

Markers related to β-cell neogenesis include insulin, PDX1, and Ngn3, which may be associated with the downstream signaling of GLP-1R/Wnt/JAK pathways (171). Puerarin mitigates endoplasmic reticulum stress-induced β-cell apoptosis by inhibiting the PERK-eIF2-ATF4-CHOP pathway in MIN6 cells damaged by H_2_O_2_, possibly mediated by the inactivation of JAK2/STAT3 signaling (172). It also protects β cells and exerts antidiabetic effects by targeting the TGF-β signaling pathway (173). Puerarin reduces β-cell apoptosis in T2DM mice by inhibiting pro-apoptotic proteins such as AIF and caspases 3, 8, and 9. TUNEL staining and pathological analyses demonstrated improvements in the degree of β-cell apoptosis and pancreatic tissue pathology (174). The direct protective effects of puerarin on β cells also involve the PI3K/Akt pathway. Puerarin alleviates cobalt chloride-induced apoptosis in MIN6 cells, thereby protecting insulin secretion levels (175). Wang et al. showed that in H_2_O_2_-induced MIN6 β cells, puerarin reduced intracellular ROS and mitochondrial superoxide levels, protecting β cells from oxidative stress. Moreover, a G6PD inhibitor counteracted the protective effects of puerarin, indicating that puerarin exerts its effects by enhancing G6PD activity (176). Puerarin can induce the polarization of RAW264.7 macrophages toward the M2 phenotype under high-glucose conditions, downregulating levels of inflammatory factors by inhibiting NF-κB and MAPK signaling pathways, thereby reducing macrophage infiltration in local tissues (177). Furthermore, in T2DM with elevated free fatty acids, macrophages regulate inflammatory responses through P2X4R. The anti-inflammatory effects of puerarin are mediated by inhibiting the P2X4R pathway, leading to reduced expression of downstream inflammatory signals, including TNF-α, iNOS, and NO, thereby providing cellular protective effects (178).

### 3.7 Other herbal monomers

Several other herbal monomers are also used to treat T2DM due to their beneficial bioactive components. For instance, maenghyeol-hwasu, an isoflavone derived from the root of the leguminous plant *Astragalus*, is a promising agent for improving β-cell apoptosis. It can inhibit apoptosis signaling in INS-1 cells induced by IL-1β, primarily by reducing the Bax/Bcl-2 ratio and caspase 3 activity and suppressing NF-κB activation and the formation of iNOS and NO (179). Besides quercetin and luteolin, other flavonoids such as apigenin, epicatechin, and rutin also exhibit protective effects on β cells (180). Mechanistically, these compounds exert antioxidant effects on mitochondria, such as reducing intracellular ROS levels, restoring mitochondrial membrane potential lost due to inflammation, and modulating KATP channels, which stimulate β-cell insulin secretion. The insulin secretion pathways may include PLC/PKC and cAMP/PKA signaling (180). Saffron is a plant from the *Iridaceae* family commonly used as a culinary spice; its active component, β-carotene, possesses antihyperglycemic, anti-inflammatory, and antioxidant pharmacological effects. Saffron extract exhibits anti-inflammatory properties by inhibiting inflammatory mediators and cytokines during diabetes, thereby improving inflammation-induced insulin resistance and reducing β-cell apoptosis by downregulating harmful molecules, such as p53 protein and caspases (181). Moreover, ginsenosides extracted from ginseng, such as Rb1, Rg1, Rg3, and Rh2, have been extensively studied for their ability to inhibit various pathways and molecular mechanisms of β-cell damage in T2DM. These include the regulation of p44/42 MAPK activation (182), activation of ERK and p38 MAPK (183), activation of PKA (184), inhibition of the Fas and caspase 3 signaling pathways (185), and modulation of cell cycle-related protein pathways like Akt/Foxo1/PDX-1 (186). Recently, Miao et al. investigated the effects of ginsenosides on diabetic endothelial function and found that ginsenoside Rk1 mitigated endothelial dysfunction and oxidative stress in diabetic mice by activating the PPAR/eNOS pathway (187).

## 4 Other T2DM treatment options and potential new therapies related to islet-cell crosstalk

**Figure 2:**
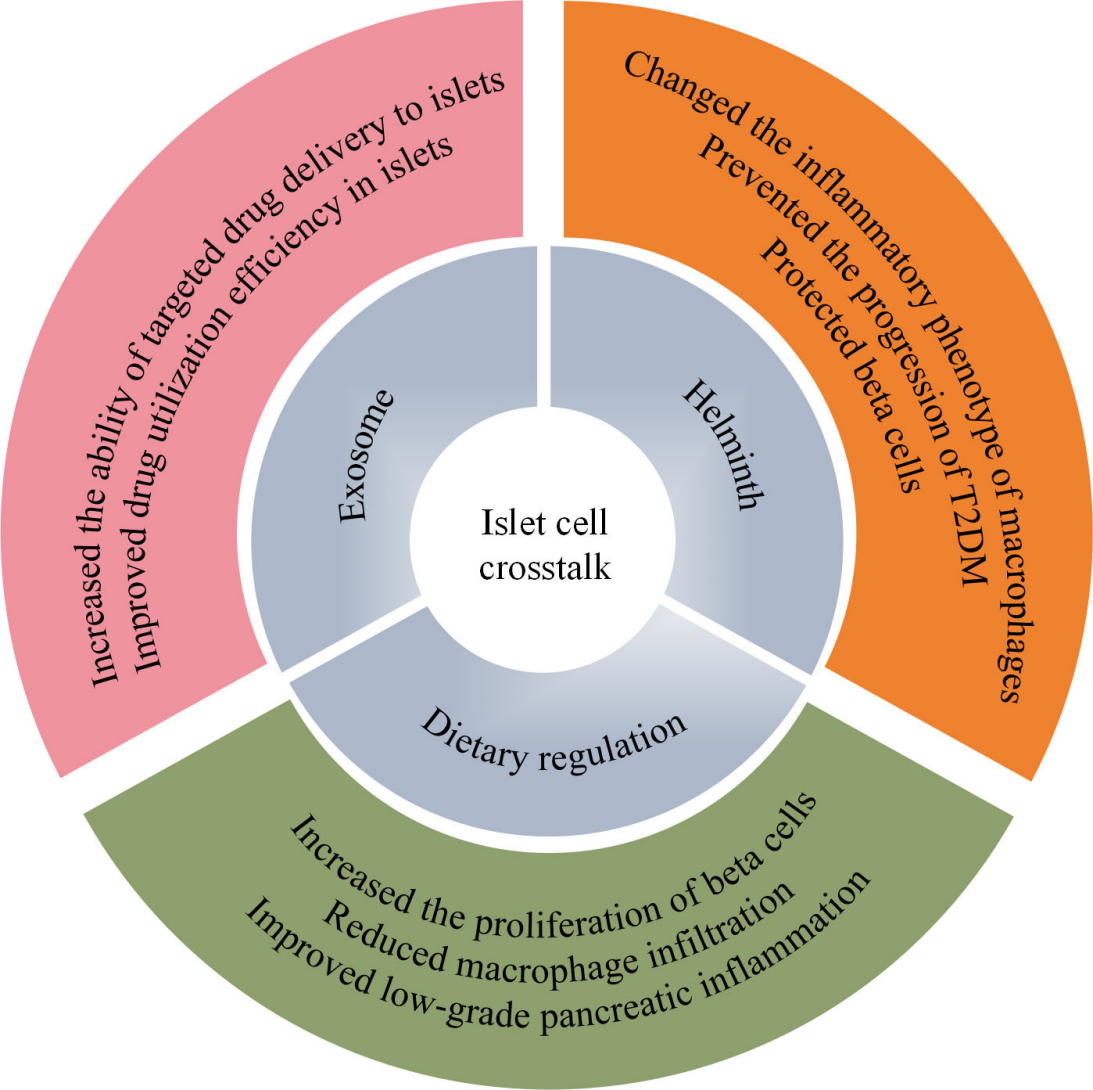
Potential personalized therapeutic mechanisms for T2DM based on islet-cell crosstalk. Personalized therapeutic approaches, including exosomes, parasites, and dietary regulation, may enhance the targeted delivery of drugs to the islets *in vivo*, modify the inflammatory phenotype of macrophages, and alleviate islet inflammation. These effects collectively protect islet β cells and support the treatment of T2DM. T2DM, type 2 diabetes mellitus.

Given the effects of herbal monomers on islet-cell crosstalk in T2DM, targeted approaches focusing on islet cell interactions can facilitate personalized treatment of T2DM. New insights into exosomes, Helminth, and dietary modulation, may offer more therapeutic options and potential new therapies for cellular crosstalk (**Figure 2**).

### 4.1 Extracellular vesicles

Despite the positive effects of phytochemicals on restoring islet-cell function, enhancing their targeted delivery and tissue utilization efficiency in islets remains essential. The International Society of Extracellular Vesicles (ISEV) recommends using “*EVs*” to describe all cell-released lipid-enclosed nanoparticles (188). These *EVs*, including exosomes and microvesicles, play key roles in intercellular communication by transferring biomolecules such as proteins and nucleic acids (*mRNA* and *miRNA*). As previously mentioned, exosomes exhibit essential auxiliary communication capabilities in islet-cell crosstalk and play a significant role in modulating intracellular signaling by binding to target cells (189), which may hold great potential for T2DM treatment. Currently, the field of exosomes and isolation techniques is developing rapidly. As potential physiological modulators and therapeutic carriers, exosomes are secreted from cells in a regulated manner, transported via the circulatory system, and exert biological effects at distant cell sites (190).

Using exosomal delivery to facilitate the transplantation of bone marrow mesenchymal stem cells (MSCs) has emerged as a new strategy in diabetes replacement therapy, protecting islets from hypoxic and pro-inflammatory factors, thereby improving the efficiency of intercellular information exchange (191). Additionally, MSCs alleviate β-cell dysfunction by modulating the inflammatory macrophage phenotype in T2DM. Recent studies have further confirmed their beneficial effects using low-dose pharmacological interventions, such as decitabine (192). Oh et al. introduced EVs derived from mouse pancreatic β-cell lines into a cellular microenvironment platform composed of bone marrow cells, islet-like cell clusters, and an extensive capillary network, successfully differentiating insulin-secreting β cells (193). Furthermore, utilizing superparamagnetic iron oxide nanoparticle-modified exosomes to carry quercetin enhanced the targeted quercetin delivery to the pancreas, effectively increasing its concentration in islets and improving its protective effects against T2DM (194). This has been validated in *in vivo* and *in vitro* studies. In summary, exosomes loaded with proteins and nucleic acids mediate the crosstalk between macrophages and β cells through intercellular transfer. They also serve as potential biomarkers for diabetes diagnosis by influencing glucose and lipid metabolism, insulin secretion, and sensitivity by regulating various molecular pathways, including AMPK, PI3K/Akt, and β-catenin (195). Another localized drug delivery method within the cellular microenvironment involves using polymer microspheres to deliver therapeutic agents.

These microspheres, carrying active substances, form hybrid clusters with islet cells and are then implanted in diabetic mice, facilitating *in situ* drug delivery and slow release within islets, thereby improving drug utilization efficiency in cellular therapies (196).

### 4.2 Helminth

Helminths typically inhabit the bodies of host mammals, prompting and regulating immune responses and tissue repair upon detection by immune cells. Notably, reports of helminth infections indicate that these helminths interact with macrophages by modulating various soluble factors and mediators. Early activated macrophages initiate a type 2 immune cascade to expel helminths (197). Through the action of helminth-derived and actively secreted molecular products—such as various proteins, glycoproteins, and lipid-like heterogeneous mixtures—host macrophages undergo functional changes and exhibit a series of bio-inflammatory effects (198). For example, in tissue damage due to helminth-mediated inflammation, recruited and activated macrophages display characteristic M2 phenotype markers, including arginase-1 (Arg1), Ym1, and resistin-like molecules (RELM) (197). In this process, M2 macrophages promote the expression of VEGF, IGF-1, MMPs, TGF-β, and PDGF, activating fibroblasts and endothelial cells, thus facilitating collagen formation and angiogenesis (199), which aid in tissue healing and inflammation control. These findings suggest that helminths can alter the phenotype and function of macrophages under pathological conditions. Moreover, helminths can impede T2DM progression by transforming the inflammatory phenotype of macrophages, thereby protecting β cells.

Previous studies have reported the beneficial effects of infections with *Heligmosomoides polygyrus*, *Trichinella* sp*iralis*, and *Schistosoma mansoni* on glucose tolerance and insulin sensitivity in obese T2DM mice, as well as the polarization, recruitment, and anti-inflammatory responses of M2-like macrophages in adipose tissue (200–203). Under infection by the helminth *Nippostrongylus brasilie*nsis *(Nippo)* or during metabolic stress induced by high-fat diets, M2-like macrophages exhibit increased IGF1R signaling. Interestingly, mice infected with *Nippo* demonstrated better insulin sensitivity than did uninfected mice. Furthermore, the ablation of IGF1R signaling in the bone marrow delays the resolution of helminth infections and predisposes to high-fat diet-induced obesity and insulin resistance. IGF1 is also crucial in the transition of pancreatic macrophages to a repair state and their secretion of factors following β-cell death (38). Additionally, secretions from the helminth parasite *Fasciola hepatica* (FhHDM-1) function as immune-modulating peptides that reduce macrophage secretion of IL-1β by inhibiting lysosomal acidification and cathepsin B-mediated NLRP3 inflammasome activation (204), a key inflammatory factor in the crosstalk between pancreatic macrophages and β cells. Overall, these findings indicate that helminth infections may influence the signaling exchanges between macrophages and β cells.

### 4.3 Dietary modulation

Excessive and inappropriate dietary habits can induce diabetes and its related complications, obesity, and associated cognitive impairments (205–207). A randomized, single-blind trial administering glucose-dependent insulinotropic peptide or saline to the participants found that a high-energy diet increased MCP-1 levels in human adipose tissue and circulation. MCP-1 is an inflammatory chemokine associated with diabetes that triggers interactions between macrophages and other cells, inducing tissue inflammation (208). This suggests that dietary strategies should be considered in cellular crosstalk to prevent the progression of metabolic diseases such as T2DM. A dietary nutrition health survey conducted in South Korea from 2007 to 2012 showed a link between dietary antioxidants and T2DM risk (209). Antioxidants can aid in the treatment of T2DM by preventing the exhaustion of pancreatic β cells and endothelial dysfunction (210). Therefore, developing a dietary pattern targeting the disease may hold therapeutic potential for β-cell function and endothelial inflammation.

In a weight management program that included dietary control, monitoring plasma MIF concentrations in participants revealed that individuals engaged in dietary control exhibited significantly lower MIF levels than did individuals with obesity without dietary and exercise interventions. This reduction is associated with improved β-cell function (211). As more food-based plants are recognized for their potential in managing T2DM through nutritional interventions, evidence supports the effectiveness of intermittent fasting and calorie control in combating T2DM and enhancing β-cell function. Luo et al. demonstrated that a traditional Chinese medicine nutritional diet comprising herbal medicine, whole grains, fruits, and vegetables significantly improved insulin secretion and glucose tolerance in diabetic mice, reduced macrophage infiltration, and increased β-cell proliferation. This effect may be linked to the modulation of gut microbiota (212). Furthermore, incorporating beneficial compounds into the diet and developing functional foods with potential therapeutic effects also contribute to improving β-cell function and preventing diabetes.

For example, a flavonoid supplement created by combining cocoa powder and carob powder prevented macrophage infiltration into the pancreas and excessive production of pro-inflammatory cytokines, as well as inactivation of NF-κB in diabetic obese rats. This intervention, when combined with metformin, demonstrated auxiliary therapeutic potential in slowing T2DM progression of T2DM (213). Additionally, a low-ketogenic diet can help reduce glucose-stimulated insulin secretion (GSIS) in insulin-sensitive rats. Furthermore, adding ketones to the diet can reverse pancreatic macrophage infiltration and IL-1β expression in diabetic rats, restoring the activity of mitochondrial respiratory chain cytochrome c oxidase and alleviating low-grade pancreatic inflammation (214).

Moreover, β-cell replacement therapy based on stem cell technology can provide a renewable source for β-cells lost due to disease. Currently, several ongoing studies and clinical trials are exploring the application of this cell therapy in T2D (215). Like traditional islet transplantation, immune suppression remains a key issue that must be addressed. To this end, researchers have developed various strategies, including genetic engineering, immune modulation, and encapsulation devices, to protect transplanted β-cells from immune system attacks. Combining these strategies helps advance the clinical translation of cell therapy for T2DM.

## 5 Future perspectives

The failure of pancreatic β cells is a critical driving factor in T2DM progression. Recent research on the interactions among β cells, islet macrophages, and endothelial cells has focused on the mediators and mechanisms regulating intercellular crosstalk; however, comprehensive analysis regarding islet-cell crosstalk in T2DM remains lacking. Additionally, most studies have utilized rodent models, the composition and function of which significantly differ from those of human islets. For example, human data indicate that the baseline proliferation rate of β cells is 10 times lower than that in mice (216). This suggests that data regarding intercellular relationships in islet cells significantly vary between animal models and humans, lacking direct evidence for human T2DM. Several key questions remain to be addressed, particularly the relationship between macrophages that are recruited to infiltrate the islets and the resident islet macrophages. The roles of these two populations during inflammation and their respective proportion remain to be explored.

The interactions among various target organs in T2DM have been extensively investigated (217, 218). Acute or chronic damage to islet tissue during the disease course is a key pathogenic factor in diabetes. The crosstalk among islet cells influences the T2DM progression. Emerging strategies to regulate intercellular communication signaling within the islets represent promising avenues for diabetes treatment. Notably, some natural compounds that target the mechanisms underlying intercellular communication among islet cells have been developed, including flavonoids and polyphenols. Some bioactive components have demonstrated favorable therapeutic effects in diabetes management. Recent mechanistic studies have revealed novel roles for natural compounds in T2DM, including the reprogramming of macrophage metabolic pathways (219), targeted modulation of the gut microbiota (220), and bitter taste-sensing type 2 receptor-mediated regulation of glucose and lipid metabolism (221). Despite incomplete elucidation of their multitarget mechanisms, the unique bioactivities of these compounds position them as promising candidates for next-generation, mechanism-based therapies. However, further studies to explore their safety, effective concentrations, and potential adverse reactions remain warranted prior to clinical application. In parallel, single-cell multi-omics will be instrumental in pinpointing cell-type-specific targets of natural products, thereby guiding the development of novel therapeutic strategies for T2DM. Additionally, targeted delivery methods for islet administration show significant potential in enhancing the effectiveness of drug delivery to islets. Exosomes or other EVs provide a natural pathway for targeted delivery, given their role in islet-cell crosstalk. Therapies involving parasites and dietary modulation also offer potential new treatment strategies for personalized medicine. These findings show that different intervention methods, including herbal monomers, are essential in diabetes treatment strategies by targeting islet-cell crosstalk.
